# Fear in the Chinese Population: Influential Patterns in the Early Stage of the COVID-19 Pandemic

**DOI:** 10.3389/fpsyg.2021.567364

**Published:** 2021-06-01

**Authors:** Beijing Chen, Xiaoxiao Sun, Fei Xie, Mengjia Zhang, Sitong Shen, Zhaohua Chen, Yuan Yuan, Peixia Shi, Xuemei Qin, Yingzhe Liu, Yuan Wang, Qin Dai

**Affiliations:** ^1^Department of Nursing Psychology, Army Medical University, Chongqing, China; ^2^Department of Clinical Nursing, Southwest Hospital of Army Medical University, Chongqing, China; ^3^Department of Neurology, Xin Qiao Hospital of Army Medical University, Chongqing, China; ^4^Department of Neurology, The Hospital of 81st Group Army PLA, Zhang Jiakou, China; ^5^Department of Teaching and Research Support Center, Army Medical University, Chongqing, China

**Keywords:** COVID-19, Chinese population, fear, pandemic, mental health

## Abstract

Major global public health emergencies challenge public mental health. Negative emotions, and especially fear, may endanger social stability. To better cope with epidemics and pandemics, early emotional guidance should be provided based on an understanding of the status of public emotions in the given circumstances. From January 27 to February 11, 2020 (during which the cases of COVID-19 were increasing), a national online survey of the Chinese public was conducted. A total of 132,482 respondents completed a bespoke questionnaire, the Emotion Regulation Questionnaire, and the Berkeley Expressivity Questionnaire (BEQ). Results showed that at the early stage of the COVID-19 epidemic, 53.0% of the Chinese population reported varying degrees of fear, mostly mild. As seen from regression analysis, for individuals who were unmarried and with a relatively higher educational level, living in city or area with fewer confirmed cases, cognitive reappraisal, positive expressivity and negative inhibition were the protective factors of fear. For participants being of older age, female, a patient or medical staff member, risk perception, negative expressivity, positive impulse strength and negative impulse strength were the risk factors for fear. The levels of fear and avoidant behavior tendencies were risk factors for disturbed physical function. Structural equation modeling suggested that fear emotion had a mediation between risk perception and escape behavior and physical function disturbance. The findings help to reveal the public emotional status at the early stage of the pandemic based on a large Chinese sample, allowing targeting of the groups that most need emotional guidance under crisis. Findings also provide evidence of the need for psychological assistance in future major public health emergencies.

## Introduction

Major public health emergencies have a profoundly negative influence on public health, which not only seriously threatens the life safety of the public, but also brings huge psychological impact to the population. The COVID-19 pandemic occurred at the beginning of 2020. The World Health Organization announced that COVID-19 is a “worldwide public health emergency” on the 30th of January 2020. By the end of May 2020, there were more than 5 million confirmed cases worldwide, with about 347 thousand losing their lives because of infection. As a result, the global social stability has been significantly endangered.

Fear is an instinctive emotional response in human beings when facing life-threatening events. During an epidemic, fear is potentially adaptive or protective for the individual (McEwen, 2007). However, over-generated fear might endanger physical function and produce negative behavioral reactions, which will have adverse effects on people’s mental health, quality of life and social stability (Su and Wei, 2005). During the Ebola outbreak in West Africa from 2013 to 2016, behaviors caused by fear were significant throughout the outbreak, leading to an increase in virus transmission, interference with effective treatment, and indirect mortality from non-Ebola diseases (Shultz et al., 2016). During the Ebola epidemic in 2016, a survey in Guangzhou of China, a city with a large number of African immigrants, showed that 31% of students reported negative emotions, such as panic, fear, and worry (Lau et al., 2016). During the SARS epidemic in 2003, as people became more aware of the seriousness of SARS, there was widespread panic and fear of going out (Wang and Luo, 2003). In the Taiwan region, fear seriously affected people’s daily lives, and there was evidence that the underground passenger flow reduced by 1,200 people for each new case (Wang, 2014). The spread of fear may lead to disturbances in physical health, e.g., during the SARS period, a student showed fever symptoms, and then 15 classmates in the same class consequently developed into fever symptoms, which was diagnosed as a mass hysteria caused by the “SARS” panic after investigation (Pu et al., 2003). These results highlighted the importance of fear during epidemics. However, there is a lack of investigations using large samples into the early public fear response during health outbreaks. Such research would be of great value in informing the development of targeted psychological interventions and providing effective psychological guidance.

Under crisis, many factors might influence the emotion of personal fear. Risk perception refers to people’s feelings and understanding about the potential risks affecting daily life, and is also an index of public panic (Sitkin and Pablo, 1992; Sitkin and Weingart, 1995). Individuals with higher risk perceptions are more likely to develop irrational tension or panic (Shi et al., 2003). For example, during the H1N1 pandemic outbreak in 2009, people who considered the severity and susceptibility of the pandemic to be higher and who concerned whether the government was well prepared were more likely to be depressed than others (Lau et al., 2010). Relatively, reasonable perception of the risks guaranteed adequate health-seeking behavior (Lau et al., 2005). However, excessive risk perception may lead to escape behaviors (Jiang et al., 2009). These results confirmed the effect of risk perception on personal emotion. However, during the current COVID-19 outbreak, it is not clear whether risk perception is a risk predictor of fear, which is important for the provision of early emotional guidance.

Emotional regulation strategies refer to the processes through which individuals exert influence on the occurrence, expression, and perception of emotions. This important coping style affects the outcome of negative events. Cognitive reappraisal and expressive suppression are two emotional regulation mechanisms that directly influence individual emotions (Ciuluvica et al., 2019). Cognitive reappraisal occurs early in the emotion-generative process and expressive suppression occurs late in the emotion-generative process (John and Gross, 2007; Cheng et al., 2009). Researchers (Gross and John, 2003) pointed out that cognitive reappraisal allows an individual to re-explain an event and change its effect on emotions. It had been reported that cognitive reappraisal could reduce negative emotions effectively (Kobayashi et al., 2020), while the effect of expressive suppression was weaker. Gross also pointed out that emotional expressivity is a kind of regulation strategy opposite to expressivity suppression, which has a unique influence on emotions, related to negative emotion and mental health problems (Gross and John, 1995). But, it had also been pointed out that the effect of expression suppression on individual emotion regulation was different in different cultural backgrounds (Liu et al., 2016). For example, in the western culture, expressivity suppression usually played a negative role (Boekaerts and Monique, 2013); while in the eastern culture, expressivity suppression may played a positive role (Dou et al., 2013). A study of Chinese college students confirmed that, expressive suppression was as effective as cognitive reappraisal in down-regulating the intensity of experienced negative emotion, and expressive suppression dampens negative emotion more quickly than cognitive reappraisal in Chinese individuals (Yuan et al., 2015). However, previous researchers have usually observed the effect of emotion regulation strategies on emotion broadly, while the protective or risk effects of different types of emotional regulation strategies on different types of emotion, and especially fear during a crisis, have not been systematically revealed.

When fear emotions persist, behavior patterns change. Studies have shown that fear emotion was related to increased avoidant behaviors (Barr et al., 2008; Smith et al., 2009). For example, during the SARS epidemic, some Taiwan nurses (especially married nurses), applied for resignation, and exhibited higher risk perceptions and stronger fear (Chong et al., 2004). Furthermore, people who were depressed during the H1N1 pandemic were more likely to take avoidant action (Lau et al., 2010). These results suggest that negative emotions might increase the avoidant behaviors.

The experience of intense or long-term stress can disrupt personal physical functions, such as loss of appetite, indigestion difficulties, and sleep problems (Chen, 2004). It has been reported that psychological responses, in particular, negative emotions including fear and depression are correlated with sleep disturbance (Zhang et al., 2003). Moreover, people’s diet behavior is influenced by emotional arousal, including fear and anger (Canetti et al., 2002). Among which, fear may lead to diet disturbance in young adult and adolescents (Anderson et al., 2018), as well as resulting in greater sleep disturbance (Fidel et al., 2018). These results suggest that negative psychological responses, especially fear, closely correlate with physical disturbance.

Previously, Myer raised a triage assessment system (TAS) for crisis circumstances. The TAS assesses personal affective, behavioral, and cognitive reactions toward crisis events (Myer and Conte, 2006). However, the TAS does not include the evaluation of physical function, which is also an important index of the acute stress response (Milligen et al., 2020). In Tong’s stress response model, panic is the most important factor in the acute stress response, followed by a defense response, and cognition of the outbreak (Tong, 2004). However, what is the relationship between fear and risk factors, avoidant behaviors, and physical disturbance? Does fear bridge a role between them? What kind of risk factors predict fear? Does fear predict avoidant behaviors and physical disturbance? The answers to these questions remain largely unknown but are crucial for the development of psychological support programs for future crises.

In the face of negative events, personal psychological responses interact with each other, and this might also be influenced by demographic variables such as gender. Many studies have shown a significant correlation between gender and fear (Egbor and Akpata, 2014; Kazancioglu et al., 2015), for example, a study showed that women are more dental surgical fear than men (Mohammed et al., 2014). It has also been reported that fear is more common in young patients (Appukuttan et al., 2015), and has a significant inverse relationship with the age of individuals (Egbor and Akpata, 2014). However, these results have been inconsistent, with evidence of higher levels of fear in older patients (Beatriz et al., 2015). In terms of marital status, there are significant differences in the scores for fear among married, unmarried, divorced, and widowed patients (Egbor and Akpata, 2014). In China, the study of fear in the recurrence of gynecologic tumors has shown that many factors, such as age, marital status, and educational level, have direct and indirect effects on fear (Qi et al., 2016; Meng et al., 2019). These results confirm the potential prediction of demographic factors relating to fear during epidemics. However, which factors are protective and which are risky remains unclear.

In sum, this study aimed to observe the status of fear in the Chinese population during the increasing stages of the COVID-19 pandemic. The trend over time, predictors from demographic variables (gender, age, degree of education, marital status, person type, and confirmed cases in city or area) and psychological variables (risk perception and emotional regulation), and the relationship with avoidant behavior tendencies and disturbed physical function, were explored through a national online investigation. Our hypotheses were: (1) Chinese people may experience fear at an early stage in the COVID-19 pandemic, and the level of fear might gradually decrease along with time; (2) demographic factors, such as gender, age, degree of education, marital status, person type, confirmed cases in city or area, might be related to the level of fear, among them, being female, older age and higher education level may increase the fear emotion, and being unmarried and having fewer confirmed cases may decrease the fear emotion; (3) risk perception and negative expressivity might increase fear emotion, while cognitive reappraisal, expressive suppression, positive expressivity, and negative inhibition might decrease fear emotion; (4) fear might increase avoidant behaviors and disturbed physical function; and (5) fear might have a bridging role between risk perception, avoidant behavior tendencies, and physical disturbance.

## Materials and Methods

### Participants

Individuals in the Chinese population aged between 18 and 75 years old, who could read and write Chinese, and were able to access a computer or smartphone with internet, were eligible for this online national investigation conducted between January 27 and February 11, 2020. Questionnaire with incomplete and invalid answers were excluded from the formal analysis. From the 135,458 collected questionnaires, 132,482 questionnaires were effective. This included 129,190 participants (97.52%) from the general population, 3,025 participants (2.28%) classified as medical staff member, 95 (0.07%) confirmed patients, 93 (0.07%) suspected patients, 36 (0.03%) recovered patients, and 43 (0.03%) family members of patients. There were more women (55.1%) than men (44.9%) and 74.8% were aged 20-49 years old. The lower education level (middle school or lower) was 56.5, and 52.8% of the population were married, and 54.5% had experienced outbreaks such as SARS. According to the confirmed cases in the city or area, respondents from cities with >10,000 cases accounted for 3.1% of the overall sample.

### Instruments

Based on previous literature and mature questionnaire at home and abroad (Chen et al., 2003; Shi et al., 2003; Wang and Luo, 2003), a self-designed questionnaire was developed by the authors, which comprised of demographic variables, fear emotion, cognitive sources of fear, risk perception section, avoidant behavior tendency section, and disturbed physical function section.

#### Demographic Variables

General information: Basic demographic characteristics, including gender, age, degree of education (middle school or lower, high school, college, and postgraduate degree or higher), marital status (married, unmarried, divorced, and widowed), person type (general population, confirmed patients, suspected patients, recovered patients, family members of patients, and medical staff member), confirmed cases in city or area (>10,000 cases, 1,000–10,000 cases, 500–1,000 cases, 100–500 cases, and <100 cases) and whether the respondent had previously experienced an outbreak such as SARS, were collected.

#### Psychological Factors

Fear emotion: one question with five options (none, mild, moderate, severe, extremely and severe/unbearable) was presented: “How much fear do you feel today?”

Cognitive sources of public fear: to explore possible cognitive sources of public fear, 14 questions were presented (with yes or no response options) relating to fear: being infected by the virus, the possibility of people being infected without isolation, new confirmed cases, death after infection, shortage of protective supplies, the possibility of people being infected without protection, death number, disrupted work or study after the pandemic, new foci, new suspected cases, insufficient cooperation of patients, being isolated due to the pandemic, insufficient duty by medical staff members, and others. The C. Hoyt’s reliability *r* was 0.251 in this study.

Risk perception: to measure people’s risk perception during the pandemic, three questions were presented (with yes or no response options): “This is a severe outbreak,” “The pandemic is close to me,” “I am in danger.” Exploratory factor analysis [EFA, principal axis factoring (PAF)] and reliability analysis showed that the KMO of the scale was 0.622, accounting for 57.94% of the total variance and Cronbach’s alpha was 0.635.

Avoidant behavior tendencies: To measure potential avoidant behavior tendencies during the pandemic, three questions were presented (with yes or no response options): “I am intending to run away if possible,” “To escape isolation, I might not go to hospital if I am a suspected case,” “To protect myself and families, I might quit the job if I am medical staff member.” The KMO of this scale was 0.687, accounting for 77.88% of the total variance, and Cronbach’s alpha was 0.857.

Disturbed physical function: to observe potential disturbed physical health under pandemic, three questions were presented (with yes or no response options): “Within the past week, I cannot keep regular schedule as usual,” “Within the past week, I cannot eat well as usual,” “Within the past week, I cannot sleep well as usual.” The KMO of this scale was 0.602, accounting for 56.20% of the total variance, and Cronbach’s alpha was 0.607.

Emotional regulation strategies: the Emotion Regulation Questionnaire consisting of (ERQ) 10 items was used (Gross and John, 2003) as translated into Chinese (Wang et al., 2007). High scores indicate higher cognitive reappraisal and expressive suppression, respectively. Cronbach’s alpha coefficient was 0.827 for cognitive reappraisal and 0.714 for expressive suppression in this study.

The Berkeley Expressivity Questionnaire (BEQ; Gross and John, 1995) was used to assess personal emotional expression. The Chinese version of BEQ comprises 16 items and five subscales (Zhao et al., 2015): positive expressivity, negative expressivity, negative inhibition, positive impulse strength, and negative impulse strength. The Cronbach’s alpha coefficient was 0.834 in this study.

### Procedures

Questions were listed in an online questionnaire, which was screened and approved by the Human Research Ethics Committee of the Army Medical University of China and Wenjuanxing online platform^1^ which providing functions equivalent to Amazon Mechanical Turk. After click-signing on an online informed consent form, individuals completed the questionnaire through an online link. The target population was the individuals under the pandemic (except special careers, such as medical workers, police, military, etc.). The questionnaire included variables about demographic information and psychological factors (fear emotion, sources of fear, risk perception, avoidant behavior tendency, disturbed physical function, emotional regulation, and emotional expressivity).

### Statistical Analysis

*T*-test and one-way ANOVA were conducted to explore the demographic characteristics of fear. *t*-test analyze was carried out to analyze the relationship between risk perception, emotional regulation strategies, and fear. χ^2^ test was carried out to observe the effects of fear emotion on avoidant behavior tendencies and disturbed physical function. Stratified linear hierarchical regression analysis was carried out to observe the predictors of fear emotion, in which demographic variables were put as first layer, and psychological factors were second layer. Linear regression analysis was also carried out to observe the prediction of fear emotion on disturbed physical function. Structural equation model was carried out with AMOS 24.0 to test the direct and mediating effect of fear emotion on avoidant behavior tendencies and disturbed physical function. Evidence of model fit was determined according to standard interpretations of the fit indices, including CFI values of at least.950, and an RMSEA no greater than.080 (Hu and Bentler, 1999). Bootstrap tests (2,000 repeated samples and 95% confidence interval) were used to test the significance of the mediating effect (Baron and Kenny, 1986), with 95% CI did not contain 0 indicating a significant mediating effect.

## Results

### Levels of Fear in the Chinese Population

In total, 70, 207 (53.0%) of the Chinese population in this study reported different degrees of fear, with a score of 1.71 ± 0.81. Through frequency analysis, it was found that 62,275 participants (47.0%) reported not experiencing fear, 50,764 participants (38.3%) reported mild fear, 15,255 participants (11.5%) reported moderate fear, 3,404 participants (2.6%) reported severe fear, and 784 participants (0.6%) reported extremely severe fear.

### Cognitive Sources of Fear

**FIGURE 1:**
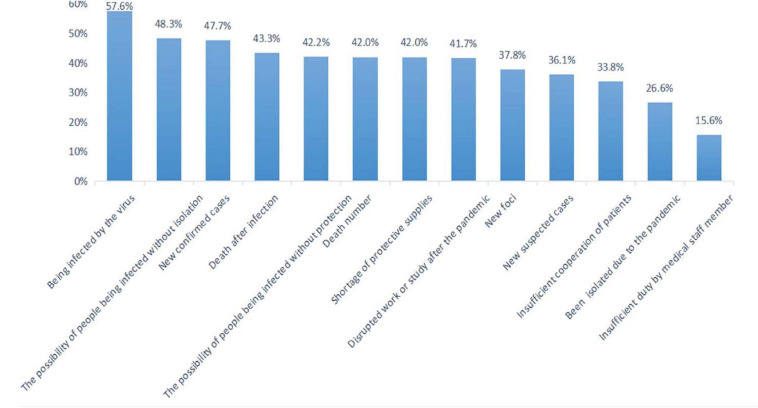
The sources of fear in Chinese population. ***P < 0.001.

Frequency analysis showed that the top three causes of fear were: being infected by the virus, the possibility of people being infected without isolation, and new confirmed cases (see Figure 1). In addition, insufficient duty by medical staff member and being isolated due to the pandemic were the bottom two reasons for fear.

### Impact of Demographic Characteristics on Fear

To observe the potential relationship between demographic characteristics and people’s fear, independent *t*-tests and one-way ANOVAs were carried out. These found that the scores for fear differed by gender, age, degree of education, marital status, person type, city type (categorized by confirmed cases in city or area), and whether having experienced epidemic such as SARS. Higher levels of fear were found in females, people of elder age, individuals with postgraduate or higher degrees, patients and medical staff member, individuals coming from a city or area with most serious levels of pandemic and those who had experienced SARS, while unmarried people reported lowest levels of fear (see Table 1).

**TABLE 1:** Comparison of fear in different variables (*N* = 1,32,482).

Variables		$\bar{x}$ ± SD	*t/F*	*P*
Gender	Male	1.68 ± 0.80	−14.590	*P* < 0.001
	Female	1.74 ± 0.82		
Age	<19	1.67 ± 0.80	35.56	*P* < 0.001
	20–29	1.66 ± 0.80		
	30–39	1.73 ± 0.82		
	40–49	1.74 ± 0.82		
	50–59	1.73 ± 0.82		
	>60	1.76 ± 0.82		
Marital status	Married	1.75 ± 0.82	88.39	*P* < 0.001
	Unmarried	1.67 ± 0.79		
	Divorced	1.72 ± 0.86		
	Widowed	1.77 ± 0.97		
Educational level	Middle school or lower	1.74 ± 0.89	78.82	*P* < 0.001
	High school	1.67 ± 0.79		
	College	1.71 ± 0.77		
	Postgraduate degree or higher	1.83 ± 0.80		
Person type	General population	1.71 ± 0.81	60.66	*P* < 0.001
	Confirmed patients	2.42 ± 1.43		
	Suspected patients	2.43 ± 1.22		
	Recovered patients	2.14 ± 1.22		
	Family members of patients	2.28 ± 1.05		
	Medical staff member	1.88 ± 0.75		
Confirmed cases in city or area	>10,000 cases	1.83 ± 0.86	33.88	*P* < 0.001
	1,000–10,000 cases	1.73 ± 0.81		
	500–1,000 cases	1.70 ± 0.80		
	100–500 cases	1.70 ± 0.82		
	<100 cases	1.70 ± 0.81		
Experienced an outbreak such as SARS	Yes	1.73 ± 0.81	9.84	*P* < 0.001
	No	1.69 ± 0.81		

### Relationship Between Fear With Risk Perception, Avoidant Behavior Tendency, Physical Function, and Emotion Regulation Strategy

#### Impact of Risk Perception and Emotion Regulation Strategy on Fear

**FIGURE 2:**
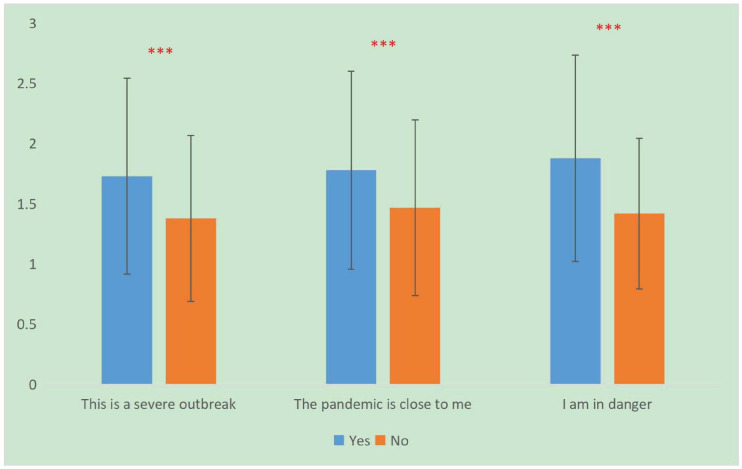
The effect of risk perception on fear in Chinese population. ^∗∗∗^*P* < 0.001.

As can be seen from Figure 2, those who indicated “This is a severe outbreak,” “The pandemic is close to me” and “I am in danger” reported highest levels of fear (*P* < 0.001) (for detailed values see Supplementary Table 1).

Through correlation analysis, we found that cognitive reappraisal (*r* = −0.010, *P* < 0.001) and expressive suppression (*r* = −0.018, *P* < 0.001) were negatively correlated with the level of fear. Positive expressivity (*r* = 0.043, *P* < 0.001), negative expressivity (*r* = 0.155, *P* < 0.001), positive impulse strength (*r* = 0.123, *P* < 0.001), and negative impulse strength (*r* = 0.180, *P* < 0.001) were positively correlated with the level of fear; and negative inhibition (*r* = −0.039, *P* < 0.001) were negatively correlated with the level of fear.

#### Impact of Fear Emotion on Avoidant Behavior Tendency and Disturbed Physical Function

**FIGURE 3:**
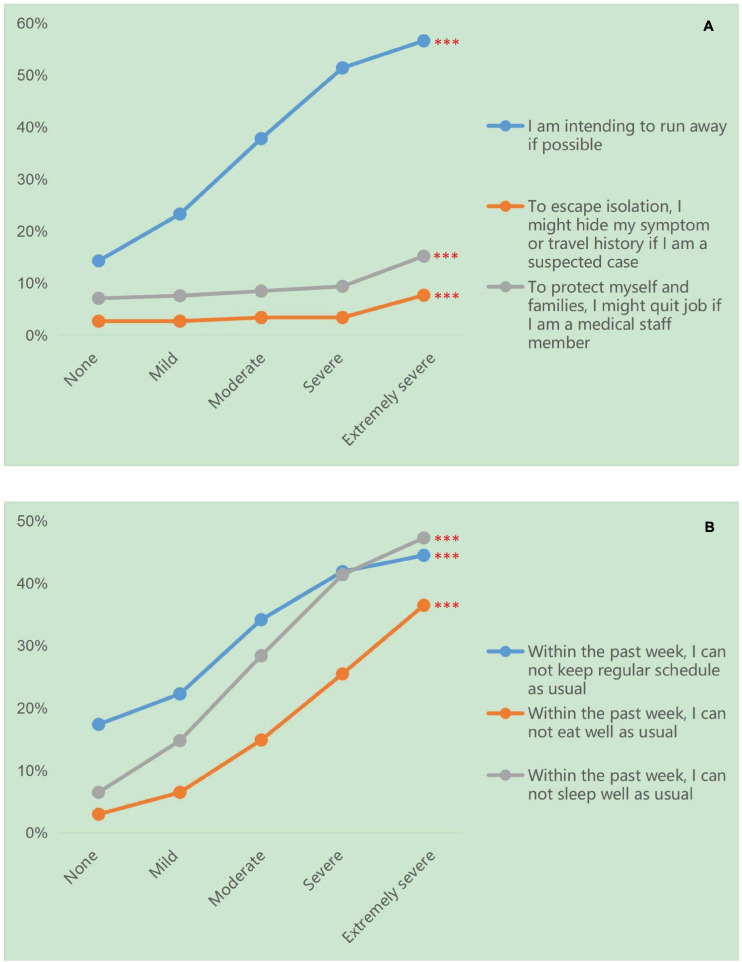
The effect of fear on avoidant behavior tendencies and disturbed physical function in Chinese population. **(A)** The effect of fear on avoidant behavior tendencies in Chinese population. **(B)** The effect of fear on disturbed physical function in Chinese population. ^∗∗∗^*P* < 0.001.

As the level of fear emotion increases, the proportion of the population “intending to run away if possible” (χ^2^ = 6762.34, *P* < 0.001, df = 4), planning “not go to hospital if I’m suspected” (χ^2^ = 94.23, *P* < 0.001, df = 4) and to “quit the job if I’m medical staff member” (χ^2^ = 118.54, *P* < 0.001, df = 4) increased (see Figure 3A).

As expected, as the level fear increased, the disturbed physical function of the population who “cannot keep regular schedule as usual” (χ^2^ = 3,112.13, *P* < 0.001, df = 4), who “cannot eat well as usual” (χ^2^ = 6219.46,*P* < 0.001, df = 4) and who “cannot sleep well as usual” (χ^2^ = 8725.31, *P* < 0.001, df = 4) increased significantly (see Figure 3B).

### Regression Analysis of Fear and Disturbed Physical Function

With the level of fear as the dependent variable, a stratified linear regression was conducted with demographic factors (including age, gender, marital status, degree of education, person type, and city type) and psychological factors (including risk perception and emotional regulation) as independent variables. The results showed that among the demographic factors, being unmarried, having a relatively higher educational level (high school and college), and living in a city or area with fewer confirmed cases were protective factors of the level of fear. Being older, female, having a postgraduate or higher educational level, being a patient or medical staff member, were risk factors for fear. Among the psychosocial factors, risk perception, negative expressivity, positive impulse strength and negative impulse strength were risk factors for the level of fear, while cognitive reappraisal, positive expressivity and negative inhibition were protective factors (Adjusted *R*^2^ = 0.105, *P* < 0.001) (see Table 2).

**TABLE 2:** Stratified regression modeling results for fear.

Predictors	Model 1		Model 2	
		
	B (95% CI)	*t*	B (95% CI)	*t*
**Demographic factors**				
Age	0.001 (0.001, 0.002)	4.014***	0.001 (0.000, 0.001)	2.646**
Gender				
Male	Ref		Ref	
Female	0.042(0.030, 0.055)	6.643***	0.035 (0.023, 0.047)	5.794***
Marital status				
Married	Ref		Ref	
Unmarried	−0.072 (−0.081, −0.063)	−15.601***	−0.028(−0.037, −0.020)	−6.432***
Divorced	−0.029 (−0.056, −0.003)	−2.143*	−0.014 (−0.039, 0.012)	−1.049
Widowed	0.009 (−0.049, 0.067)	0.306	0.039 (−0.016, 0.094)	1.397
Educational level				
Middle school or lower	Ref		Ref	
High school	−0.067 (−0.079, −0.056)	−11.382***	−0.049 (−0.060, −0.038)	−8.629***
College	−0.039 (−0.050, −0.028)	−7.057***	−0.027 (−0.038, −0.017)	−5.082***
Postgraduate degree or higher	0.073 (0.048, 0.098)	5.767***	0.092 (0.068, 0.116)	7.569***
Person type				
General population	Ref		Ref	
Confirmed patients	0.698 (0.536, 0.861)	8.409***	0.569 (0.415, 0.723)	7.238***
Suspected patients	0.695 (0.531, 0.860)	8.280***	0.519 (0.363, 0.675)	6.535***
Recovered patients	0.422 (0.157, 0.686)	3.127**	0.267 (0.017, 0.517)	2.092*
Family members of patients	0.564 (0.322, 0.806)	4.571***	0.401 (0.172, 0.629)	3.433**
Medical staff member	0.151 (0.121, 0.180)	9.991***	0.047 (0.019, 0.075)	3.289**
Confirmed cases in city or area				
>10,000 cases	Ref		Ref	
1,000–10,000 cases	−0.105 (−0.131, −0.079)	−7.944***	−0.066 (−0.090, −0.041)	−5.284***
500–1,000 cases	−0.132 (−0.157, −0.106)	−10.019***	−0.093 (−0.117, −0.069)	−7.478***
100–500 cases	−0.140 (−0.166, −0.113)	−10.372***	−0.092 (−0.117, −0.067)	−7.244***
<100 cases	−0.152 (−0.184, −0.120)	−9.236***	−0.095 (−0.126, −0.065)	−6.116***
**Psychological factors**				
Risk perception				
This is a severe outbreak				
No			Ref	
Yes			0.135 (0.115, 0.156)	13.043***
The pandemic is close to me				
No			Ref	
Yes			0.081 (0.070, 0.093)	13.872***
I am in danger				
No			Ref	
Yes			0.392 (0.383, 0.402)	81.580***
Emotion regulation				
Cognitive reappraisal			−0.004 (−0.004, −0.003)	−9.308***
Expressive suppression			−0.001 (−0.002, 0.000)	−1.932
Positive expressivity			−0.013 (−0.015, −0.011)	−15.266***
Negative expressivity			0.010 (0.009, 0.012)	14.718***
Negative inhibition			−0.003(−0.005, −0.001)	−3.671***
Positive impulse strength			0.005(0.004, 0.007)	6.032***
Negative impulse strength			0.034 (0.032, 0.035)	39.248***
Adjusted *R*^2^	0.008		0.113	
F(df_1_, df_2_), *p*-value	*F*(17, 1,32,464) = 65.26, *P* < 0.001		*F*(27, 1,32,454) = 626.51, *P* < 0.001	

*P < 0.05, **P < 0.01, ***P < 0.001.

With disturbed physical function as the dependent variable, regression analysis showed that the level of fear and avoidant behavior tendencies were risk factors for disturbed physical function (Adjusted *R*^2^ = 0.074, *P* < 0.001) (see Table 3).

**TABLE 3:** Linear regression modeling results for disturbed physical function.

Predictors	Model 1	
	
	B (95% CI)	*t*
Fear emotion		
None	Ref	
Mild	0.155 (0.146, 0.164)	34.145***
Moderate	0.473 (0.459, 0.486)	68.475***
Severe	0.766 (0.740, 0.792)	57.143***
Extremely severe/unbearable	0.942 (0.889, 0.996)	34.561***
Avoidant behavior tendencies	0.130 (0.123, 0.138)	33.523***
Adjusted *R*^2^	0.074	
F(df_1_, df_2_), *p*-value	F(5, 1,32,476) = 2132.38, *P* < 0.001	

***P < 0.001.

### Mediation Analysis of Fear Emotion

Confirmatory factor analysis of risk perception, escape behavior tendency, and physical function disturbance were carried out (see Supplementary Figure 1), which confirmed that path coefficients for each model were significant (*P* < 0.001).

**FIGURE 4:**
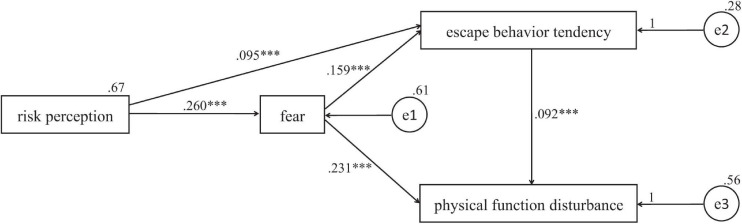
Mediation analysis of fear emotion. ^∗∗∗^*P* < 0.001.

To further explore the interaction between fear emotion and risk perception, avoidant behavior tendency, and physical function, a hypothesis-driven model test was carried out as Figure 4. The model fit showed that each index of the model was good (χ^2^/df = 7.65, GFI = 1.00, AGFI = 1.00, RMSEA = 0.007), which indicated that risk perception had a positive direct effect on fear emotion and avoidant behavior tendency, and indirect effect on avoidant behavior tendency (0.041, 0.04–0.043) and disturbed physical function (0.073, 0.073–0.075). Fear emotion had positive direct effect on avoidant behavior tendency and disturbed physical function, and indirect effect on disturbed physical function (0.015, 0.014–0.017). The results indicated a positive effect of risk perception on fear emotion, and a mediation effect of fear emotion between risk perception and avoidant behavior tendency and disturbed physical function.

## Discussion

This study observed fear in the Chinese population at an early stage of the COVID-19 outbreak and its relationship with risk perception, avoidant behavior tendencies, physical function, and emotional regulation through a large sample (*N* = 1,32,482) online national investigation. The findings showed that the Chinese population experienced some fear, but was not panicking during the pandemic. Being unmarried, having a relatively high educational level, living in a city or area with fewer confirmed cases, cognitive reappraisal, positive expressivity and negative inhibition were protective predictors of the level of fear. Being of older age, female, having a postgraduate or higher educational level, being a patient or medical staff member, risk perception, negative expressivity, and positive/negative impulse strength were risk predictors of the level of fear.

### Level of Fear in the Chinese Population

In this study, 53.0% of the Chinese population reported a degree of fear, indicating that fear was prevalent during the outbreak. Further analysis found that this fear was mainly mild, indicating that the Chinese population was not panicking. This finding helps the Chinese government and international organizations better understand the Chinese population’s emotional status under the COVID-19 pandemic.

### Cognitive Sources of Fear in the Chinese Population

The top three sources of fear in this study were: being infected by the virus, the possibility of people being infected without isolation, and new confirmed cases. During the SARS epidemic, fear came mainly from the characteristics of SARS (strong infectivity and high risk) and the temporary lack of effective treatment (Chen et al., 2003). It can be seen that the characteristics of the disease itself (infectious and high risk) were the main source of fear in public. Findings confirmed that the main sources of fear came from both the possible influence of the pandemic on the individual, and the macro-development of a national epidemic. These findings provide suggestions to the government about required emotional guidance during the pandemic, i.e., knowledge education and information for the public.

### Influential Factors of Fear

Our study showed that there were stronger levels of fear in females and people of older age. Older participants may have experienced stronger fear due to having poorer health status and being more vulnerable to the virus. This is consistent with the fact that unmarried young people reported the lowest levels of negative emotion due to most likely having better health status in general. Individuals with postgraduate or higher degrees and people who had experienced SARS may have had stronger fear because they knew more about the dangers of viruses. As hypothesized, people from Hubei province (an area with most serious pandemic levels) reported stronger fear. As expected, patients and medical staff members reported the strongest fear, especially patients who were confirmed and suspected cases. However, recovered patients reported relatively lower fear, which was consistent with reporting regarding SARS (Tse et al., 2003). These results allow us to identify the populations that most need emotional guidance, and to focus limited psychological resources during epidemics.

In this investigation, participants who thought “This is a severe outbreak,” “The pandemic is close to me,” and “I am in danger” had higher levels of fear. This “cognitive fear” (Song et al., 2018) increased the fear levels significantly. Therefore, early and reasonable risk perception interventions are particularly important when adjusting fear in the Chinese population. This study have shown that cognitive reappraisal, expressive suppression and negative inhibition were negatively correlated with fear emotion; while positive expressivity, negative expressivity, positive/negative impulse strength were positively correlated with fear emotion. In Chinese culture, expressive suppression was not entirely an inappropriate regulation strategy. East Asian culture emphasized avoiding hurting others and striving to maintain harmonious relationships, suppression was associated with better social functioning (Butler et al., 2007; Soto et al., 2011; Yuan et al., 2015). For example, a study of insurance workers in Hong Kong showed that the increase of suppression was associated with fewer negative emotions (Yeung and Fung, 2012). Another study showed that Asian-Americans who rated suppression as more valuable had better emotional responses to anger elicited (Mauss and Butler, 2010). Similarly, among Chinese college students, the relationship between suppression and interpersonal harmony was significantly positive (Su et al., 2012; Wei et al., 2013). Thus, when people experience fear emotion during an epidemic, they can modulate it through the selection of an emotional regulation strategy, i.e., greater cognitive reappraisal and expressive suppression and less expression are recommended.

### Prediction of Demographic Factors and Psychological Factors for Fear

As seen from the regression analysis, we could see that being unmarried, having a relatively high educational level, and living in a city or area with fewer confirmed cases were protective factors for fear. Being of older age, female, having studied at the postgraduate or higher educational level, being a patient or medical staff members were risk factors. After controlling for demographic factors, cognitive reappraisal, positive expressivity and negative inhibition were protective factors for fear, and risk perception, negative expressivity, and positive/negative impulse strength were risk factors. This study systematically explored the protective and risk predictors of fear taking into account demographic and psychological variables. These findings will help focus on specific populations most in need of future psychological interventions and offer further evidence to support the development of more effective psychological training programs.

### Prediction of Fear and Avoidant Behavior Tendency on Physical Function

We found that fear increased avoidant behavior tendencies and significantly disturbed physical function. This is consistent with a large number of studies, confirming that negative emotions are associated with a poor lifestyle, such as sleep and diet (Huang et al., 2013; Zhu et al., 2016; Cai et al., 2019; Li et al., 2019). Regression analysis indicated that the level of fear and avoidant behavior tendencies positively predicted disturbed physical function. The results suggested a bridging role of fear between risk perception, avoidant behaviors, and physical disturbance. Thus, to better maintain normal psychological and physical function under crisis, intervention, and guidance on fear emotion is critical. Previously, the TAS for crisis intervention provided a framework for understanding clients’ reactions during a crisis. This investigation broadened TAS theory through the inclusion of disturbed physical health, and helped to develop more targeted and directed early intervention to prevent these problems.

### Limitations

First, fear in the population was only assessed using one subjective item, and there was a lack of systematic objective evaluation. Second, there was a lack of in-depth exploration of the impact factors of fear, such as psychological resilience, coping style, and so on. Third, this was a cross-sectional study, which precludes causal conclusions. However, with a large sample that covered all provinces and areas of China, this study was sufficiently powerful to accurately reflect public fear in China during the COVID-19 pandemic. Moreover, this online investigation was carried out during the case increasing stage of the pandemic (from January 27 to February 11, 2020), which allowed clear observation regarding the trends of fear during this period.

## Conclusion

At the early stage of the epidemic, the Chinese public experienced a mild degree of fear which declined over time. Fear functions as a bridge between risk perception, avoidant behaviors, and physical disturbance. The protective factors (being unmarried, having a relatively high educational level, living in city or area with fewer confirmed cases, cognitive reappraisal, positive expressivity, and negative inhibition) and risk factors (being of older age, female, having a postgraduate or higher educational level, being a patient or medical staff member, risk perception, negative expressivity, and positive/negative impulse strength) for fear suggest that the government could establish a long-term psychological stress monitoring mechanism to grasp the psychological dynamics of the public under major emergencies in a timely way and provide effective psychological interventions. The current snapshot of public emotion offers theoretical evidence for psychological assistance and emotional guidance during a crisis, and provides suggestions as to how best to deliver psychological support in future major public health emergencies.

## Data Availability Statement

The raw data supporting the conclusions of this article will be made available by the authors, without undue reservation.

## Ethics Statement

The studies involving human participants were reviewed and approved by the Human Research Ethics Committee of the Army Medical University of China and Wenjuanxing online platform (www.wjx.top). The participants provided their written informed consent to participate in the study.

## Author Contributions

QD designed the study, collected the data, and reviewed and revised the manuscript. BC collected and analyzed the data and final manuscript. XS and FX assisted the data collection and contributed to the manuscript writing. MZ, SS, ZC, YY, PS, XQ, YL, and YW assisted the data collection. All authors have read and approved the submitted version.

## Conflict of Interest

The authors declare that the research was conducted in the absence of any commercial or financial relationships that could be construed as a potential conflict of interest.

## Footnotes

1 www.wjx.top
