# Supplementary material for: Fear in the Chinese Population: Influential Patterns in the Early Stage of the COVID-19 Pandemic
Source: Front Psychol. 2021 Jun 1;12:567364. doi: 10.3389/fpsyg.2021.567364 (PMC8204111; doi:10.3389/fpsyg.2021.567364)
Supplement: Supplementary file 1 [file Table_1.DOCX]

**Supplementary materials**

**Table S1 The effect of risk perception on fear（N=132482）**

| **Variables** |  | **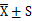**±S.D.** | ***t*** | ***P*** |
| --- | --- | --- | --- | --- |
| This is a severe outbreak | Yes | 1.73±0.81 | 33.42 | *P*＜0.001 |
|  | No | 1.38±0.68 |  |  |
| The pandemic is close to me | Yes | 1.78±0.82 | 55.66 | *P*<0.001 |
|  | No | 1.47±0.73 |  |  |
| I am in danger | Yes | 1.88±0.86 | 103.85 | *P*<0.001 |
|  | No | 1.42±0.63 |  |  |
