# Supplementary figures and images for: Fear in the Chinese Population: Influential Patterns in the Early Stage of the COVID-19 Pandemic
Source: Front Psychol. 2021 Jun 1;12:567364. doi: 10.3389/fpsyg.2021.567364 (PMC8204111; doi:10.3389/fpsyg.2021.567364)

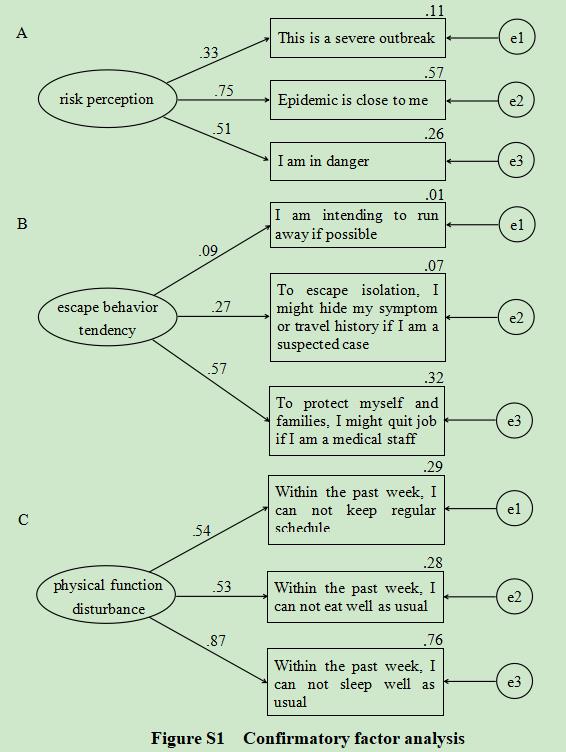

Supplement: Supplementary file 2 [file Image_1.jpg]
